# Development, prevention, and treatment of feeding tube dependency

**DOI:** 10.1007/s00431-017-2908-x

**Published:** 2017-04-13

**Authors:** Hilde Krom, J. Peter de Winter, Angelika Kindermann

**Affiliations:** 1 Emma Children’s Hospital, Box 22666, 1100 DD Amsterdam, The Netherlands; 2 Spaarne Hospital, Box 770, 2130 AT Hoofddorp, The Netherlands

**Keywords:** Feeding disorders, Avoidant/restrictive food intake disorder, Tube feeding, Tube dependency, Tube weaning, Clinical hunger provocation

## Abstract

Enteral nutrition is effective in ensuring nutritional requirements and growth. However, when tube feeding lasts for a longer period, it can lead to tube dependency in the absence of medical reasons for continuation of tube feeding. Tube-dependent children are unable or refuse to start oral activities and they lack oral skills. Tube dependency has health-, psychosocial-, and economy-related consequences. Therefore, the transition to oral feeding is of great importance. However, this transition can be very difficult and needs a multidisciplinary approach. Most studies for treatment of tube dependency are based on behavioral interventions, such as family therapy, individual behavior therapy, neuro-linguistic programming, and parental anxiety reduction. Furthermore, oral motor therapy and nutritional adjustments can be helpful in tube weaning. The use of medication has been described in the literature. Although mostly chosen as the last resort, hunger-inducing methods, such as the Graz-model and the Dutch clinical hunger provocation program, are also successful in weaning children off tube feeding.

*Conclusion*: The transition from tube to oral feeding is important in tube-dependent children but can be difficult. We present an overview for the prevention and treatment of tube dependency.

**Table:** 

**What is known:** • *Longer periods of tube feeding can lead to tube dependency.* *• Tube weaning can be very difficult.*
**What is new:** • *Weaning as soon as possible and therefore referral to a multidisciplinary team are recommended.* • *An overview of treatment options for tube dependency is presented in this article.*

## Introduction

Eating and drinking are needed to sustain life and ensure growth [36]. Therefore, enteral nutrition might be needed when the child is unable to eat, malnourished, or when dietary measures are insufficient to fulfill nutritional requirements [18]. Although effective in these situations, when tube feeding lasts for a longer period, it can lead to difficulties in the transition to normal oral feeding and tube dependency when these children are medically stable [2, 28, 39].

Tube dependency is an unintended result of long-term enteral feeding [13] and is defined as the active refusal to eat (or drink), lack of motivation or inability to learn, or showing no precursors of eating development and skills after long-term enteral feeding [12, 15, 28]. The child consequently remains dependent on the feeding tube, although there are no medical grounds for the continuation of tube feeding anymore [12, 13].

Common knowledge about the normal feeding process and development is inevitable to understand how a difficult transition to oral feeding in tube-fed children may arise. Feeding gradually matures from reflexes in newborns to a voluntary act at the age of 6–7 months [18]. The normal feeding process (hunger-satiation system) consists of three phases: (1) the pre-oral phase in which the child feels hungry, leading to appetite and nutritional intake; (2) the oro-pharyngeal phase in which the foods are prepared orally, transported from tongue to pharynx, and swallowed; and (3) the gastro-intestinal phase in which satiation and digestion occur [23, 32]. A normal feeding process requires a complex interaction between physiological (medical) factors (especially the cardiac, respiratory and digestive tracts are important), sensorimotor functions, and parental and pediatric factors [18, 23].

Problems in any of these separate factors can attribute to a disruption of the normal feeding development in tube-fed children [23, 28], but it may be the subtle interaction between those factors that causes some of them to remain tube dependent for a long term [28]. In general, tube dependency arises from a decreased motivation to eat due to a poor perception of hunger and to satiation by tube feeding, negative experiences (such as nausea, vomiting, esophagitis, and nasogastric or endotracheal tube replacement) leading to oral aversion, an impaired child-caregiver interaction, and reduced positive oral stimulation (lack of experience). Tube placement at a young age, receiving tube feeding between the sensitive period for feeding skills and interest for new foods, and the duration of the tube feeding are risk factors for this development [15, 28].

The introduction of enteral nutrition, improved techniques, and advanced medical care has contributed to the survival of infants and children who would not have survived years ago. These children have a greater risk of medical complications interfering with the infant’s feeding, and many are fed by tube feeding. Preterm infants, infants who have chronic medical conditions, infants with tube feeding between the ages 3 and 6 months, and children with long-term tube feeding, are more at risk of developing severe feeding disorders [6, 32]. Therefore, tube-dependent children are usually fragile but medically stable survivors of neonatal intensive care or child surgery [2, 13, 35]. Unfortunately, no data regarding the prevalence and/or incidence of tube dependency is available, probably due to the fact that tube dependency is not recognized as a separate disorder in medical classification systems such as the International Statistical Classification of Diseases and Related Health Problems (ICD) or the Diagnostic and Statistical Manual of Mental Disorders (DSM) in the psychiatric field. Tube feeding dependency, however, should be seen as a severe feeding disorder. In pediatric medical literature, different definitions and classifications are used for feeding disorders [5, 22]. Tube-dependent children may now fulfill the criteria for “avoidant/restrictive food intake disorder” (ARFID) (see Table 1) according to the DSM-5 [1]. This diagnosis replaces and extends the previously used diagnosis “feeding disorder of infancy and early childhood” of the DSM-IV-TR [22, 29], which was not sufficient for tube-dependent children when “significant weight loss” or “failure to gain weight” was prevented by the tube feeding [30].

**Table 1:** Criteria DSM-5: avoidant/restrictive food intake disorder (ARFID) [1]

A. An eating or feeding disturbance (e.g., apparent lack of interest in eating or food, avoidance based on the sensory characteristics of food, concern about aversive consequences of eating) as manifested by persistent failure to meet appropriate nutritional and/or energy needs associated with one (or more) of the following:
- Significant weight loss (or failure to achieve expected weight gain or faltering growth in children)
- Significant nutritional deficiency
- Dependence on enteral feeding or oral nutritional supplements
- Marked interference with psychosocial functioning
B. The disturbance is not better explained by lack of available food or by an associated culturally sanctioned practice.
C. The eating disturbance does not occur exclusively during the course of anorexia nervosa or bulimia nervosa, and there is no evidence of a disturbance in the way in which one’s body weight or shape is experienced.
D. The eating disturbance is not attributable to a concurrent medical condition or not better explained by another mental disorder. When the eating disturbance occurs in the context of another condition or disorder, the severity of the eating disturbance exceeds that routinely associated with the condition or disorder and warrants additional clinical attention.
Specify if: in remission: after full criteria for avoidant/restrictive food intake disorder were previously met, the criteria have not been met for a sustained period of time.

Besides the absence of a medical reason for continuing enteral feeding in tube dependency, long-term tube feeding may also have serious side effects and complications, such as infections, aspiration, airway blocking, severe feeding disorders, and interaction problems between caregivers and child (see Table 2) [12, 13, 33]. Parents of children with feeding disorders often suffer from feeding-related anxiety, and mothers may show greater attachment insecurity [8, 9]. Dunitz-Scheer reported that the quality of life of these infants and their families is severely affected [12] and Wright described a highly distressing situation for these parents [40]. In addition, tube feeding is expensive [40]. As a consequence, tube dependency has significant health-, psychosocial-, and economy-related effects [3]. Therefore, the prevention of tube dependency and tube weaning methods is of great importance.

**Table 2:** Side effects and complications of (long-term) tube feeding [12, 13, 33]

Feeding disorders
Selectivity
Tube dependency
Oppositional and aversive behavior
Dysfunctional feeding situations
Interaction problems between parents and child
Gagging
Recurrent daily vomiting
Discomfort
Oversensitivity
Dumping syndrome
Skin eczema
Perforations
Infections
Dislocations
Leakage
Blockage
Aspiration
Airway blocking

## Prevention of tube dependency

Several recommendations to prevent tube dependency and to provide normal feeding in tube-fed children can be addressed, mostly based on the normal hunger-satiation system. Goals and strategies of the tube feeding should be defined and assessed in each patient, and tube weaning should be discussed when goals are achieved [12]. Underlying problems such as cow’s milk allergy or gastroesophageal reflux disease should be diagnosed and treated properly to avoid aversive experiences [12, 28]. Aiming to maintain the normal hunger-satiation system and stimulate oral activity and intake, oral feeding supplemented by tube feeding boluses or nocturnal tube feeding can be given. Early oral normalization programs should be offered and supervised by a speech-language pathologist or an occupational therapist [12, 16, 33]. Oral feeding skills can be practiced by nonintrusive sucking [12, 33]. The child should be involved in the mealtime environment with all sensory stimulations of foods (sight, smell, and sound) and has an adequate social interaction with family members [18, 28, 33]. When the child is on boluses, the tube feeding should be given at that moment. Forced feeding and urged feeding should be avoided and forbidden [12]. When long-term tube feeding is expected, a percutaneous endoscopic gastrostomy (PEG) could be considered to reduce negative oral experiences and promote positive oral experiences [12, 18, 28].

## Tube weaning methods

The transition from tube to oral feeding (weaning) may take only several days or weeks, but in others, it can be very challenging, lasting months to years [2, 18, 28, 39]. Since weaning can be very difficult, it is best accomplished with a multidisciplinary approach, including health care workers in the field of pediatrics, dietetics, psychology, speech-language pathology, and/or occupational therapy [15, 31, 38]. The pediatrician is responsible for the overall medical well-being of the child and will take care of medical problems, diagnostics, consultations, and medication. Furthermore, in most teams, the pediatrician coordinates the feeding team [15, 38]. The dietician monitors the nutritional intake including micronutrients and anthropometrics and will assist in developing a weaning plan [15, 18]. The psychologist determines the psychological and behavioral aspects of both child and their parents concerning feeding including cultural expectations, mealtime behaviors, and comorbid psychiatric diagnoses and can implement behavioral therapies [15, 38]. The speech-language pathologist will assess whether a child has the skills to feed itself safely and will therefore evaluate the oral motor feeding skills including swallowing. Furthermore, the speech-language pathologist can implement an oral motor program to improve swallowing or chewing coordination, treat feeding-related maladaptive behavior, and improve oral tolerance [15, 18]. In some feeding teams, an occupational therapist evaluates aspects concerning oral sensory, oral motor, and positioning of the child [15].

Multidisciplinary interventions for feeding disorders consist of parental teaching, nutritional, oral motor, sensory integration therapy, and behavioral and other psychological interventions [3, 15, 26, 33]. In most studies, tube dependency is treated with behavioral interventions, such as family-based therapy, individual behavior therapy, neuro-linguistic programming, and parental anxiety reduction [3, 7, 25, 26, 34, 40]. Behavioral interventions are helpful in increasing oral intake and reducing fear of swallowing after a period of enteral feeding [3]. Several other behavioral strategies can be used: structured meals, social modeling, and positive reinforcement [14]. Attractive looking meals and favorite tastes, seeing other people eating (for instance other children at the daycare), and being in a room where meals normally occur can increase the intake [28].

Besides behavioral interventions, stimulating appetite is essential for tube weaning [14]. Some appetite-inducing methods are also mentioned in the paragraph “Prevention of tube dependency” since these can be used both as prevention and treatment for tube weaning. Both boluses at daytime and continuous feeding at night can be used to stimulate appetite. When the child is on boluses at daytime, food should be offered orally before delivering the tube feeding to stimulate oral intake. On the other hand, feeding the child continuously by tube at night may stimulate oral intake during daytime. Another beneficial effect of nocturnal tube feeding is that the child is less aware of the tube feeding. When the child accepts some food orally, the total caloric intake of the tube feeding, which the child receives in 24 h, can be reduced to stimulate oral intake [28].

When earlier mentioned methods have not been successful, patients can be referred to a more intensive weaning program. Several studies use reducing or discontinuing tube feeding for appetite stimulation as a weaning method [4, 7, 20, 21, 39]. Various hunger-inducing weaning programs exist internationally, which may be home based [26, 38] or clinical [4, 7, 20]. However, there is limited literature regarding the efficacy and safety of these programs [17].

## Hunger-inducing weaning programs

Hunger-inducing methods for tube weaning are based on hunger induction given the fact that tube feeding reduces the child’s motivation to eat and drink. The control center for appetite is located in the hypothalamus, and appetite is stimulated by energy intake reduction, which will lead to feeding in healthy children within a few hours. Hunger requires a complex interaction of sensory input, limbic and cortical modulators, visceral feedback, and hormonal effects [37]. Rapid weaning of the tube feeding stimulates the experience of hunger, which is required to cure oral feeding aversion. Creating and stimulating hunger is the drive to start eating. This process is thought to become more complicated when tube dependency exists for a longer period [20].

One of the published models is the so-called Graz-model, which is based on two principles: inducing hunger by gradually reducing tube-fed volume (physical) and strengthening the child’s autonomy (psychodynamic). Individual and group interventions occur within a 3-week inpatient program (so-called eating schools). The child will be exposed to attractive small colorful dishes and the child’s autonomy is supported without any kind of forced feeding and is as nondirective as possible [12]. Recently, this model was also implemented in a web-based method (net coaching). No significant differences were found between patients (aged 0.21–23.65 years) regarding the success of complete weaning between net coaching (90.5%) and the inpatient program (81.3%) [27].

Another hunger-inducing program is the Dutch so-called clinical hunger provocation program, which contains an inpatient tube weaning program during the period of 2–3 weeks [20, 24]. This is supported by a multidisciplinary team consisting of a pediatric gastroenterologist, speech-language pathologist, dietician, clinical psychologist child life specialist, and specialized nurses. During the first step, 50% of the normal tube feeding is given by tube by boluses. During the second step, oral feeding is offered by a nurse before the tube feeding boluses. During steps 3 and 4, respectively, tube feeding and insensible loss are given at night. Parents can offer the oral feeding when the child has started to eat. In a pilot study, ten children (aged 9–21 months; mean 15.7 months) started to eat within the first week. After 6 months, 80% were still eating adequately and gaining weight without tube feeding [24]. Based on these findings, a new, randomized controlled crossover study of 22 patients (aged 11–26 months; mean 16.3 months) was performed. This study showed an 86% (*n* = 18/21) efficacy (successful weaning) in the hunger provocation group (*P* < 0.001) compared to 9% (*n* = 1/11) in the control group (outpatient treatment by the same multidisciplinary team and tube feeding reduction of 20–25%) after 6 months. All these patients also showed weight gain during this follow-up period [20].

A German study analyzed the follow-up for 1–3 years (median 2 years) after rapid home-based weaning (4–10 days) of tube-dependent children (aged 5–57 months; median 16 months). In this program, hunger is induced by gradually reducing the total original fluids and nutrition in 5 days until 50% of original amount is achieved; after which, tube feeds are completely ceased (or reduced to minimally indicated minimal amount). At follow-up, 84.6% (*n* = 33/39) of patients were fully tube free, 5.1% needed a tube for fluids or medication only, and 10.2% were still tube fed [39]. To our knowledge, no other studies analyzing long-term effects of hunger-inducing programs are published so far.

## Medication

There is no data of how frequent medication is used to wean off tube feeding. Literature on this topic is scarce [19].

A gastrostomy tube weaning program in the USA used cyproheptadine as appetite stimulant in combination with a multidisciplinary approach 19 days during inpatient program in which tube feeds were also reduced. At discharge, 90% and after a year 83% of 30 patients (ages 3.9 ± 1.4 years) had discontinued gastrostomy tube feedings [4].

Davis et al. described a pain rehabilitation model in their 14 weeks during outpatient program in which tricyclic antidepressant (ig amitriptyline) and/or gabapentin, and appetite stimulant megestrol were prescribed. They hypothesized that food refusal could be the result of abdominal discomfort which is worsened by eating, due to pain nerve sensitization with hyperalgesia and allodynia in medically fragile toddlers [10]. In addition, tube feeding was progressively reduced and all nine children were successfully (receiving 100% of their intake orally) weaned [10]. However, a more recent randomized controlled trial of Davis et al. showed all patients transitioned to oral feeding regardless of group assignment (placebo or amitriptyline), suggesting that amitriptyline is not necessary for the transition [11].

A case report described two 24-month-old twin girls with food refusal and fear of feeding, who started to eat 2 months after the start of fluoxetine (an SSRI) to target anxiety and fear of feeding [8].

Another case series of three patients with feeding disorders showed an increased oral intake and weight gain due to risperidone, after which enteral support was discontinued in two patients and reduced in one patient [19].

We do not recommend the use of medication for tube weaning so far as limited evidence on pharmacologic interventions is available and due to possible side effects, such as CNS depression, sleep disturbances, drowsiness, palpitations, respiratory infections, gastro-intestinal complaints, and anticholinergic effects.

## Conclusion and recommendations

Tube dependency does have health-, psychosocial-, and economic-related consequences. Therefore, prevention is of great importance, and when tube dependency does develop, weaning as soon as possible is recommended.

Several treatment options are available. Most studies are based on behavioral interventions and may include family therapy, individual behavior therapy, neuro-linguistic programming, and parental anxiety reduction. Other therapies exist of nutritional modifications, oral sensorimotor skill, or sensory integration therapy. Hunger-inducing methods are very effective on short term in children with tube dependency, but long-term effects should be further investigated.

We advise referral to a multidisciplinary feeding team, with extensive experience in this field to find the most optimal treating approach for the tube-dependent child. We do not recommend the use of medication due to limited evidence and possible side effects.

## Abbreviations

ARFID: Avoidant/restrictive food intake disorder

## References

[1] American Psychiatric Association (2013) Diagnostic and statistical manual of mental disorders (5th edition). Washington

[2] Bazyk S (1990). Factors associated with the transition to oral feeding in infants fed by nasogastric tubes. Am J Occup Ther.

[3] Benoit D, Wang EE, Zlotkin SH (2000). Discontinuation of enterostomy tube feeding by behavioral treatment in early childhood: a randomized controlled trial. J Pediatr.

[4] Brown J, Kim C, Lim A, Brown S, Desai H, Volker L (2014). Successful gastrostomy tube weaning program using an intensive multidisciplinary team approach. J Pediatr Gastroenterol Nutr.

[5] Bryant-Waugh R (2013). Feeding and eating disorders in children. Curr Opin Psychiatry.

[6] Burklow KA, McGrath AM, Valerius KS, Rudolph C (2002). Relationship between feeding difficulties, medical complexity, and gestational age. Nutr Clin Pract.

[7] Byars KC, Burklow KA, Ferguson K, O'Flaherty T, Santoro K, Kaul A (2003). A multicomponent behavioral program for oral aversion in children dependent on gastrostomy feedings. J Pediatr Gastroenterol Nutr.

[8] Celik G, Diler RS, Tahiroglu AY, Avci A (2007). Fluoxetine in posttraumatic eating disorder in two-year-old twins. J Child Adolesc Psychopharmacol.

[9] Chatoor I, Ganiban J, Hirsch R, Borman-Spurrell E, Mrazek DA (2000). Maternal characteristics and toddler temperament in infantile anorexia. J Am Acad Child Adolesc Psychiatry.

[10] Davis AM, Bruce AS, Mangiaracina C, Schulz T, Hyman P (2009). Moving from tube to oral feeding in medically fragile nonverbal toddlers. J Pediatr Gastroenterol Nutr.

[11] Davis AM, Dean K, Mousa H, Edwards S, Cocjin J, Almadhoun O, et al. (2016) A randomized controlled trial of an outpatient protocol for transitioning children from tube to oral feeding: no need for amitriptyline. J Pediatr 10.1016/j.jpeds.2016.02.013 PMC4846510 26947568

[12] Dunitz-Scheer M, Levine A, Roth Y, Kratky E, Beckenbach H, Braegger C, et al. (2009) Prevention and treatment of tube dependency in infancy and early childhood. Infant Child Adolesc Nutr 73–82

[13] Dunitz-Scheer M, Marinschek S, Beckenbach H, Kratky E, Hauer A, Scheer P (2011). Tube dependence: a reactive eating behavior disorder. Infant, Child, & Adolescent Nutrition.

[14] Edwards S, Davis AM, Bruce A, Mousa H, Lyman B, Cocjin J, et al. (2015) Caring for tube-fed children: a review of management, tube weaning, and emotional considerations. JPEN J Parenter Enteral Nutr 10.1177/0148607115577449 25791833

[15] Edwards S, Davis AM, Ernst L, Sitzmann B, Bruce A, Keeler D (2015). Interdisciplinary strategies for treating oral aversions in children. JPEN J Parenter Enteral Nutr.

[16] Fucile S, Gisel E, Lau C (2002). Oral stimulation accelerates the transition from tube to oral feeding in preterm infants. J Pediatr.

[17] Gardiner AY, Fuller DG, Vuillermin PJ (2014). Tube-weaning infants and children: a survey of Australian and international practice. J Paediatr Child Health.

[18] Gottrand F, Sullivan PB (2010). Gastrostomy tube feeding: when to start, what to feed and how to stop. Eur J Clin Nutr.

[19] Gross P, Coletti DJ, Hirschkorn K, Terranova E, Simpser EF (2004). The effectiveness of risperidone in the treatment of three children with feeding disorders. J Child Adolesc Psychopharmacol.

[20] Hartdorff CM, Kneepkens CM, Stok-Akerboom AM, van Dijk-Lokkart EM, Engels MA, Kindermann A (2015). Clinical tube weaning supported by hunger provocation in fully-tube-fed children. J Pediatr Gastroenterol Nutr.

[21] Ishizaki A, Hironaka S, Tatsuno M, Mukai Y (2013). Characteristics of and weaning strategies in tube-dependent children. Pediatr Int.

[22] Kerzner B, Milano K, MacLean WC, Berall G, Stuart S, Chatoor I (2015). A practical approach to classifying and managing feeding difficulties. Pediatrics.

[23] Kindermann A, Kneepkens CMF (2010). Voedings- en eetproblemen bij jonge kinderen. Praktische Pediatrie.

[24] Kindermann A, Kneepkens CM, Stok A, van Dijk EM, Engels M, Douwes AC (2008). Discontinuation of tube feeding in young children by hunger provocation. J Pediatr Gastroenterol Nutr.

[25] Linscheid TR (2006). Behavioral treatments for pediatric feeding disorders. Behav Modif.

[26] Lukens CT, Silverman AH (2014). Systematic review of psychological interventions for pediatric feeding problems. J Pediatr Psychol.

[27] Marinschek S, Dunitz-Scheer M, Pahsini K, Geher B, Scheer P (2014). Weaning children off enteral nutrition by netcoaching versus onsite treatment: a comparative study. J Paediatr Child Health.

[28] Mason SJ, Harris G, Blissett J (2005). Tube feeding in infancy: implications for the development of normal eating and drinking skills. Dysphagia.

[29] Norris ML, Katzman DK (2015). Change is never easy, but it is possible: reflections on avoidant/restrictive food intake disorder two years after its introduction in the DSM-5. J Adolesc Health.

[30] Piazza CC, Roane HS (2009) Assessment of pediatric feeding disorders. In: Matson JL AF, Matson ML (eds). Springer

[31] Rommel N, De Meyer AM, Feenstra L, Veereman-Wauters G (2003). The complexity of feeding problems in 700 infants and young children presenting to a tertiary care institution. J Pediatr Gastroenterol Nutr.

[32] Rudolph CD, Link DT (2002). Feeding disorders in infants and children. Pediatr Clin N Am.

[33] Schauster H, Dwyer J (1996). Transition from tube feedings to feedings by mouth in children: preventing eating dysfunction. J Am Diet Assoc.

[34] Senez C, Guys JM, Mancini J, Paz Paredes A, Lena G, Choux M (1996). Weaning children from tube to oral feeding. Childs Nerv Syst.

[35] Sevilla WM, McElhanon B (2016) Optimizing transition to home enteral nutrition for pediatric patients. Nutr Clin Pract 10.1177/0884533616673348 27756847

[36] Sharp WG, Jaquess DL, Morton JF, Herzinger CV (2010). Pediatric feeding disorders: a quantitative synthesis of treatment outcomes. Clin Child Fam Psychol Rev.

[37] Shea S, Stein AD, Basch CE, Contento IR, Zybert P (1992). Variability and self-regulation of energy intake in young children in their everyday environment. Pediatrics.

[38] Silverman AH (2010). Interdisciplinary care for feeding problems in children. Nutr Clin Pract.

[39] Wilken M, Cremer V, Berry J, Bartmann P (2013). Rapid home-based weaning of small children with feeding tube dependency: positive effects on feeding behaviour without deceleration of growth. Arch Dis Child.

[40] Wright C (2013). Helping children stop or avoid enteral feeding. BMJ Qual Improv Rep.

